# 1-(4-Methoxy­phen­yl)-3-phenyl-1*H*-pyrazol-5-amine

**DOI:** 10.1107/S1600536809015463

**Published:** 2009-04-30

**Authors:** Isuru R. Kumarasinghe, Victor J. Hruby, Gary S. Nichol

**Affiliations:** a Department of Chemistry, The University of Arizona, 1306 E. University Boulevard, Tucson, AZ 85721, USA

## Abstract

The synthesis of the title compound, C_16_H_15_N_3_O, is regiospecific and single-crystal X-ray diffraction provides the only means of unambiguous structural analysis, with the benzene ring bonded to the imine C atom. The phenyl ring and the essentially planar (r.m.s. deviation 0.0354 Å) methoxy­benzene group are rotated by 29.41 (5) and 37.01 (5)°, respectively, from the central pyrazole ring. An inter­molecular N—H⋯N hydrogen bond links symmetry-related mol­ecules into a *C*(5) chain, which runs parallel to the *b* axis.

## Related literature

For background to this study, see: Gavrin *et al.* (2007 ▶); Joshi *et al.* (1979 ▶); Michaux & Charlier (2004 ▶); Ossipov *et al.* (2004 ▶); Raffa (2001 ▶).
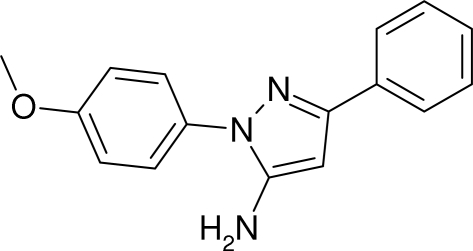


## Experimental

### 

#### Crystal data

C_16_H_15_N_3_O
*M* *_r_* = 265.31
Orthorhombic, 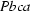

*a* = 14.9638 (6) Å
*b* = 6.3639 (2) Å
*c* = 28.2466 (12) Å
*V* = 2689.87 (18) Å^3^
*Z* = 8
Mo *K*α radiation
μ = 0.09 mm^−1^
*T* = 296 K
0.35 × 0.35 × 0.05 mm

#### Data collection

Bruker Kappa APEXII diffractometer
Absorption correction: none
11632 measured reflections
3019 independent reflections
2256 reflections with *I* > 2σ(*I*)
*R*_int_ = 0.031

#### Refinement

*R*[*F* ^2^ > 2σ(*F* ^2^)] = 0.044
*wR*(*F* ^2^) = 0.111
*S* = 1.02
3019 reflections
190 parameters
H atoms treated by a mixture of independent and constrained refinement
Δρ_max_ = 0.20 e Å^−3^
Δρ_min_ = −0.15 e Å^−3^

### 

Data collection: *APEX2* (Bruker, 2007 ▶); cell refinement: *SAINT* (Bruker, 2007 ▶); data reduction: *SAINT*; program(s) used to solve structure: *SHELXTL* (Sheldrick, 2008 ▶); program(s) used to refine structure: *SHELXTL*; molecular graphics: *ORTEP-3 for Windows* (Farrugia, 1997 ▶) and *Mercury* (Macrae *et al.*, 2008 ▶); software used to prepare material for publication: *SHELXTL* and local programs.

## Supplementary Material

Crystal structure: contains datablocks I, global. DOI: 10.1107/S1600536809015463/lh2810sup1.cif

Structure factors: contains datablocks I. DOI: 10.1107/S1600536809015463/lh2810Isup2.hkl

Additional supplementary materials:  crystallographic information; 3D view; checkCIF report

**Fig. 1.**
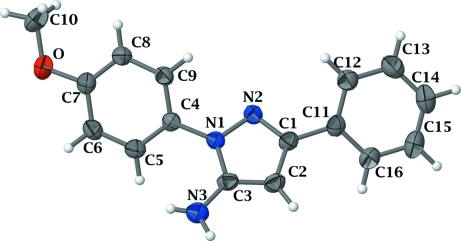
The molecular structure of (I) with displacement ellipsoids at the 50% probability level.

Enhanced figure: interactive version of Fig. 1

## Figures and Tables

**Table 1:** Hydrogen-bond geometry (Å, °)

*D*—H⋯*A*	*D*—H	H⋯*A*	*D*⋯*A*	*D*—H⋯*A*
N3—H3*NA*⋯N2^i^	0.89 (2)	2.37 (2)	3.228 (2)	162.5 (16)

Symmetry code: (i) 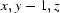.

**Fig. 2.**
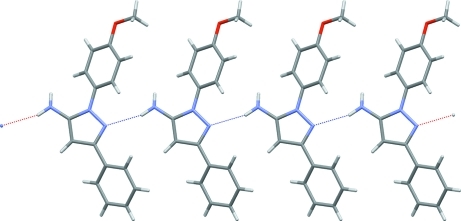
Part of the crystal structure of (I) showing a hydrogen bonded (dashed lines) chain.

## supplementary crystallographic information

### Comment

It has been pharmacologically shown that co-administration of opioids with cyclooxygenase (COX) inhibitors is synergistic and reduces development of drug tolerance (Raffa, 2001; Ossipov *et al.*, 2004). The creation of bifunctional molecules having both COX2 inhibitory activity and Opioid mu agonist activity is a promising approach to retain better analgesic properties. We are investigating plausible COX2 pharmacophores that can be obtained for bifunctional design using known organic reactions. Based on the evidence so far (Joshi *et al.*,1979; Gavrin *et al.*, 2007) we probed whether the reaction between hydrazine derivatives and benzoylacetonitrile will usefully yield pyrazoles as a product substituted with 1,5-vicinal diaryls, which is an important structural feature for COX2 inhibitory activity (Michaux & Charlier, 2004). We observed that the reaction between benzoylacetonitrile with 2-(4-methoxyphenyl)hydraziniumchloride is regiospecific which means it provides solely 1-(4-methoxyphenyl)-3-phenyl-1*H*-pyrazol-5-amine, (I), which is undesired for our purpose. Furthermore it cannot be correctly identified with one-dimensional nuclear Overhauser effect or two-dimensional heteronuclear multiple bond correlation spectroscopic methods, leaving single-crystal X-ray diffraction as the only possible means of unambiguous identification of the compound.

The molecular structure of (I) is shown in Figure 1. Two regioisomers were possible and here the structure is unambiguous with the phenyl ring (C11-C16) bonded to the imine carbon atom C1. Molecular dimensions are unexceptional. The methoxybenzyl group is essentially planar (r.m.s. deviation of a mean plane fitted through C4 to C10 and O is 0.0354 Å) and is rotated by 37.01 (5)° from the central pyrazole ring. The phenyl ring (C11-C16) is rotated by 29.41 (5)° from the central pyrazole ring. A lone N–H···N hydrogen bond links the molecules into a C(5) chain which runs parallel to the *b *axis(Figure 2).

### Experimental

Benzoylacetonitrile (1.45 g, 10 mmol) in 50 ml of absolute ethanol was treated with 2-(4-methoxyphenyl) hydraziniumchloride (1.75 g, 10 mmol) under reflux for 1 day. The solvent was then removed by rotary evaporation and the residue was extracted with a 1:1 mix of dichloromethane and H_2_O. The organic layer was separated and dried with anhydrous MgSO_4_, filtered and then the solvent removed by rotary evaporation to leave a vrown residue. This was washed with hexane and recrystallized from dichloromethane. C_16_H_15_N_3_O, (I), yield = 90%. TOF MS EI m/*z* 264.9839. ^1^H NMR (CDCl_3_)δ 3.85 (s, 3H), 5.95 (s, 1H), 7.0 (d,2*H*), 7.25 (m,1*H*), 7.4 (t,2*H*) 7.5(d,2*H*), 7.8 (d,2*H*) ^13^CNMR (CDCl_3_) δ 55.50, 87.59, 114.61, 125. 522, 126.049, 127.626, 128.391, 131.463,133.551, 145.740, 151.043, 158.971

### Refinement

Amine hydrogen atoms were freely refined. Aryl hydrogen atoms were refined with *U*_iso_(H) = 1.2 *U*_eq_(C) and a fixed C–H distance of 0.93 Å; methyl hydrogen atoms were refined with *U*_iso_(H) = 1.5 *U*_eq_(C) and a fixed C–H distance of 0.96 Å.

### Crystal data

**Table:**

C_16_H_15_N_3_O	*F*(000) = 1120
*M**_r_* = 265.31	*D*_x_ = 1.310 Mg m^−^^3^
Orthorhombic, *P**b**c**a*	Mo *K*α radiation, λ = 0.71073 Å
Hall symbol: -P 2ac 2ab	Cell parameters from 2475 reflections
*a* = 14.9638 (6) Å	θ = 2.7–28.6°
*b* = 6.3639 (2) Å	µ = 0.09 mm^−^^1^
*c* = 28.2466 (12) Å	*T* = 296 K
*V* = 2689.87 (18) Å^3^	Plate, colourless
*Z* = 8	0.35 × 0.35 × 0.05 mm

### Data collection

**Table:**

Bruker Kappa APEXII diffractometer	2256 reflections with *I* > 2σ(*I*)
Radiation source: sealed tube	*R*_int_ = 0.031
graphite	θ_max_ = 28.3°, θ_min_ = 2.0°
φ and ω scans	*h* = −19→16
11632 measured reflections	*k* = −7→6
3019 independent reflections	*l* = −36→37

### Refinement

**Table:**

Refinement on *F*^2^	Primary atom site location: structure-invariant direct methods
Least-squares matrix: full	Secondary atom site location: difference Fourier map
*R*[*F*^2^ > 2σ(*F*^2^)] = 0.044	Hydrogen site location: difference Fourier map
*wR*(*F*^2^) = 0.111	H atoms treated by a mixture of independent and constrained refinement
*S* = 1.02	*w* = 1/[σ^2^(*F*_o_^2^) + (0.048*P*)^2^ + 0.671*P*] where *P* = (*F*_o_^2^ + 2*F*_c_^2^)/3
3019 reflections	(Δ/σ)_max_ = 0.001
190 parameters	Δρ_max_ = 0.20 e Å^−^^3^
0 restraints	Δρ_min_ = −0.15 e Å^−^^3^

### Special details

**Table:**

Geometry. All e.s.d.'s (except the e.s.d. in the dihedral angle between two l.s. planes) are estimated using the full covariance matrix. The cell e.s.d.'s are taken into account individually in the estimation of e.s.d.'s in distances, angles and torsion angles; correlations between e.s.d.'s in cell parameters are only used when they are defined by crystal symmetry. An approximate (isotropic) treatment of cell e.s.d.'s is used for estimating e.s.d.'s involving l.s. planes.
Refinement. Refinement of *F*^2^ against ALL reflections. The weighted *R*-factor *wR* and goodness of fit *S* are based on *F*^2^, conventional *R*-factors *R* are based on *F*, with *F* set to zero for negative *F*^2^. The threshold expression of *F*^2^ > σ(*F*^2^) is used only for calculating *R*-factors(gt) *etc*. and is not relevant to the choice of reflections for refinement. *R*-factors based on *F*^2^ are statistically about twice as large as those based on *F*, and *R*- factors based on ALL data will be even larger.

### Fractional atomic coordinates and isotropic or equivalent isotropic displacement parameters (Å^2^)

**Table:**

	*x*	*y*	*z*	*U*_iso_*/*U*_eq_	
O	0.37170 (8)	0.3584 (2)	0.56552 (3)	0.0537 (3)	
N1	0.38199 (8)	0.10523 (19)	0.75399 (4)	0.0327 (3)	
N2	0.37654 (8)	0.2595 (2)	0.78830 (4)	0.0357 (3)	
N3	0.39632 (11)	−0.2693 (2)	0.74725 (6)	0.0506 (4)	
H3NA	0.3945 (12)	−0.386 (3)	0.7646 (6)	0.057 (6)*	
H3NB	0.3982 (15)	−0.264 (4)	0.7192 (8)	0.083 (8)*	
C1	0.38472 (9)	0.1542 (2)	0.82878 (5)	0.0334 (3)	
C2	0.39560 (10)	−0.0607 (3)	0.82140 (5)	0.0401 (4)	
H2	0.4031	−0.1639	0.8444	0.048*	
C3	0.39302 (9)	−0.0890 (2)	0.77325 (5)	0.0359 (3)	
C4	0.37681 (9)	0.1656 (2)	0.70563 (4)	0.0317 (3)	
C5	0.42957 (9)	0.0699 (3)	0.67137 (5)	0.0391 (4)	
H5	0.4681	−0.0386	0.6797	0.047*	
C6	0.42468 (10)	0.1361 (3)	0.62484 (5)	0.0414 (4)	
H6	0.4586	0.0688	0.6018	0.050*	
C7	0.36981 (9)	0.3015 (3)	0.61242 (5)	0.0379 (4)	
C8	0.31728 (9)	0.3982 (2)	0.64632 (5)	0.0374 (3)	
H8	0.2803	0.5097	0.6381	0.045*	
C9	0.32044 (9)	0.3268 (2)	0.69274 (4)	0.0347 (3)	
H9	0.2840	0.3887	0.7155	0.042*	
C10	0.32253 (15)	0.5386 (4)	0.55199 (6)	0.0687 (6)	
H10A	0.2604	0.5175	0.5591	0.103*	
H10B	0.3296	0.5620	0.5186	0.103*	
H10C	0.3442	0.6587	0.5691	0.103*	
C11	0.38252 (9)	0.2644 (3)	0.87483 (5)	0.0354 (3)	
C12	0.33514 (10)	0.4489 (3)	0.88095 (5)	0.0421 (4)	
H12	0.3041	0.5073	0.8556	0.050*	
C13	0.33371 (12)	0.5473 (3)	0.92489 (6)	0.0534 (4)	
H13	0.3018	0.6714	0.9289	0.064*	
C14	0.37933 (12)	0.4620 (3)	0.96249 (6)	0.0567 (5)	
H14	0.3784	0.5285	0.9918	0.068*	
C15	0.42634 (12)	0.2785 (3)	0.95670 (5)	0.0550 (5)	
H15	0.4571	0.2207	0.9822	0.066*	
C16	0.42822 (11)	0.1790 (3)	0.91320 (5)	0.0448 (4)	
H16	0.4601	0.0546	0.9096	0.054*	

### Atomic displacement parameters (Å^2^)

**Table:**

	*U*^11^	*U*^22^	*U*^33^	*U*^12^	*U*^13^	*U*^23^
O	0.0695 (8)	0.0595 (9)	0.0320 (5)	0.0105 (6)	0.0029 (5)	0.0047 (5)
N1	0.0413 (6)	0.0248 (8)	0.0320 (5)	−0.0005 (5)	−0.0026 (4)	−0.0017 (4)
N2	0.0451 (7)	0.0306 (8)	0.0315 (5)	−0.0003 (5)	−0.0004 (5)	−0.0021 (5)
N3	0.0764 (10)	0.0275 (9)	0.0478 (8)	−0.0004 (7)	−0.0067 (7)	−0.0014 (7)
C1	0.0349 (7)	0.0311 (9)	0.0342 (6)	−0.0036 (6)	0.0004 (5)	0.0033 (6)
C2	0.0495 (8)	0.0308 (10)	0.0399 (7)	−0.0004 (7)	−0.0037 (6)	0.0071 (6)
C3	0.0386 (7)	0.0260 (9)	0.0430 (7)	−0.0005 (6)	−0.0046 (5)	0.0011 (6)
C4	0.0347 (7)	0.0290 (9)	0.0315 (6)	−0.0037 (6)	−0.0026 (5)	0.0003 (5)
C5	0.0389 (7)	0.0379 (10)	0.0406 (7)	0.0068 (6)	−0.0009 (6)	−0.0013 (6)
C6	0.0425 (8)	0.0448 (11)	0.0370 (7)	0.0064 (7)	0.0045 (6)	−0.0061 (6)
C7	0.0418 (7)	0.0405 (10)	0.0315 (6)	−0.0032 (6)	−0.0007 (5)	−0.0002 (6)
C8	0.0427 (8)	0.0324 (9)	0.0370 (7)	0.0053 (6)	−0.0031 (5)	−0.0006 (6)
C9	0.0373 (7)	0.0327 (9)	0.0342 (6)	0.0017 (6)	0.0012 (5)	−0.0048 (6)
C10	0.0913 (15)	0.0668 (16)	0.0481 (10)	0.0174 (12)	−0.0012 (9)	0.0197 (9)
C11	0.0380 (7)	0.0355 (10)	0.0328 (6)	−0.0062 (6)	0.0033 (5)	0.0032 (6)
C12	0.0463 (8)	0.0394 (10)	0.0406 (7)	0.0001 (7)	0.0015 (6)	0.0012 (7)
C13	0.0587 (10)	0.0483 (12)	0.0532 (9)	0.0046 (8)	0.0083 (7)	−0.0089 (8)
C14	0.0659 (11)	0.0678 (15)	0.0363 (8)	−0.0051 (10)	0.0071 (7)	−0.0104 (8)
C15	0.0639 (11)	0.0683 (15)	0.0329 (7)	−0.0030 (9)	−0.0023 (7)	0.0052 (8)
C16	0.0525 (9)	0.0436 (11)	0.0382 (7)	0.0026 (8)	0.0003 (6)	0.0046 (7)

### Geometric parameters (Å, °)

**Table:**

O—C7	1.3736 (16)	C7—C8	1.3834 (19)
O—C10	1.415 (2)	C8—H8	0.9300
N1—N2	1.3820 (16)	C8—C9	1.3885 (18)
N1—C3	1.3607 (19)	C9—H9	0.9300
N1—C4	1.4211 (16)	C10—H10A	0.9600
N2—C1	1.3307 (17)	C10—H10B	0.9600
N3—H3NA	0.89 (2)	C10—H10C	0.9600
N3—H3NB	0.79 (2)	C11—C12	1.382 (2)
N3—C3	1.363 (2)	C11—C16	1.392 (2)
C1—C2	1.393 (2)	C12—H12	0.9300
C1—C11	1.4782 (19)	C12—C13	1.391 (2)
C2—H2	0.9300	C13—H13	0.9300
C2—C3	1.373 (2)	C13—C14	1.374 (2)
C4—C5	1.3896 (19)	C14—H14	0.9300
C4—C9	1.377 (2)	C14—C15	1.373 (3)
C5—H5	0.9300	C15—H15	0.9300
C5—C6	1.3823 (19)	C15—C16	1.383 (2)
C6—H6	0.9300	C16—H16	0.9300
C6—C7	1.380 (2)		
			
C7—O—C10	117.63 (13)	C7—C8—C9	119.26 (14)
N2—N1—C3	111.85 (11)	H8—C8—C9	120.4
N2—N1—C4	118.61 (12)	C4—C9—C8	120.95 (13)
C3—N1—C4	129.54 (12)	C4—C9—H9	119.5
N1—N2—C1	103.86 (12)	C8—C9—H9	119.5
H3NA—N3—H3NB	126 (2)	O—C10—H10A	109.5
H3NA—N3—C3	113.9 (12)	O—C10—H10B	109.5
H3NB—N3—C3	120.3 (18)	O—C10—H10C	109.5
N2—C1—C2	112.11 (12)	H10A—C10—H10B	109.5
N2—C1—C11	121.01 (14)	H10A—C10—H10C	109.5
C2—C1—C11	126.87 (13)	H10B—C10—H10C	109.5
C1—C2—H2	127.1	C1—C11—C12	121.62 (13)
C1—C2—C3	105.89 (13)	C1—C11—C16	119.26 (14)
H2—C2—C3	127.1	C12—C11—C16	119.10 (14)
N1—C3—N3	123.63 (13)	C11—C12—H12	119.9
N1—C3—C2	106.28 (13)	C11—C12—C13	120.14 (15)
N3—C3—C2	130.03 (15)	H12—C12—C13	119.9
N1—C4—C5	121.32 (13)	C12—C13—H13	119.9
N1—C4—C9	119.27 (12)	C12—C13—C14	120.28 (17)
C5—C4—C9	119.38 (12)	H13—C13—C14	119.9
C4—C5—H5	120.1	C13—C14—H14	120.1
C4—C5—C6	119.90 (14)	C13—C14—C15	119.89 (15)
H5—C5—C6	120.1	H14—C14—C15	120.1
C5—C6—H6	119.8	C14—C15—H15	119.8
C5—C6—C7	120.38 (13)	C14—C15—C16	120.38 (15)
H6—C6—C7	119.8	H15—C15—C16	119.8
O—C7—C6	115.73 (13)	C11—C16—C15	120.20 (16)
O—C7—C8	124.18 (14)	C11—C16—H16	119.9
C6—C7—C8	120.09 (13)	C15—C16—H16	119.9
C7—C8—H8	120.4		
			
C3—N1—N2—C1	0.13 (14)	C10—O—C7—C8	5.6 (2)
C4—N1—N2—C1	−179.13 (11)	C5—C6—C7—O	178.30 (14)
N1—N2—C1—C2	0.31 (15)	C5—C6—C7—C8	−1.9 (2)
N1—N2—C1—C11	179.87 (11)	O—C7—C8—C9	179.71 (14)
N2—C1—C2—C3	−0.62 (17)	C6—C7—C8—C9	−0.1 (2)
C11—C1—C2—C3	179.85 (13)	N1—C4—C9—C8	176.33 (13)
N2—N1—C3—N3	176.99 (14)	C5—C4—C9—C8	−1.6 (2)
N2—N1—C3—C2	−0.51 (16)	C7—C8—C9—C4	1.8 (2)
C4—N1—C3—N3	−3.9 (2)	N2—C1—C11—C12	29.1 (2)
C4—N1—C3—C2	178.65 (12)	N2—C1—C11—C16	−151.79 (14)
C1—C2—C3—N1	0.65 (16)	C2—C1—C11—C12	−151.35 (15)
C1—C2—C3—N3	−176.62 (16)	C2—C1—C11—C16	27.7 (2)
N2—N1—C4—C5	141.21 (14)	C1—C11—C12—C13	179.32 (14)
N2—N1—C4—C9	−36.72 (18)	C16—C11—C12—C13	0.3 (2)
C3—N1—C4—C5	−37.9 (2)	C11—C12—C13—C14	0.0 (3)
C3—N1—C4—C9	144.17 (15)	C12—C13—C14—C15	−0.2 (3)
N1—C4—C5—C6	−178.27 (13)	C13—C14—C15—C16	0.1 (3)
C9—C4—C5—C6	−0.3 (2)	C14—C15—C16—C11	0.1 (3)
C4—C5—C6—C7	2.1 (2)	C1—C11—C16—C15	−179.39 (15)
C10—O—C7—C6	−174.60 (16)	C12—C11—C16—C15	−0.3 (2)

### Hydrogen-bond geometry (Å, °)

**Table:**

*D*—H···*A*	*D*—H	H···*A*	*D*···*A*	*D*—H···*A*
N3—H3NA···N2^i^	0.89 (2)	2.37 (2)	3.228 (2)	162.5 (16)

Symmetry codes: (i) *x*, *y*−1, *z*.

## References

[bb1] Bruker (2007). *APEX2 *and *SAINT* Bruker AXS Inc., Madison, Wisconsin, USA.

[bb2] Farrugia, L. J. (1997). *J. Appl. Cryst.* **30**, 565.

[bb3] Gavrin, L. K., Lee, A., Provencher, B. A., Massefski, W. W., Huhn, S. D., Ciszewski, G. M., Cole, D. C. & McKew, J. C. (2007). *J. Org. Chem.*, **72**, 1043–1046. 10.1021/jo062120g 17253833

[bb4] Joshi, K. C., Pathak, V. N. & Garg, U. (1979). * J. Heterocycl. Chem.* **16**, 1141–1145.

[bb5] Macrae, C. F., Bruno, I. J., Chisholm, J. A., Edgington, P. R., McCabe, P., Pidcock, E., Rodriguez-Monge, L., Taylor, R., van de Streek, J. & Wood, P. A. (2008). *J. Appl. Cryst.* **41**, 466–470.

[bb6] Michaux, C. & Charlier, C. (2004). *Mini-Rev. Med. Chem* **4**, 603–615. 10.2174/1389557043403756 15279594

[bb7] Ossipov, M. H., Lai, J., King, T., Vanderah, T. W., Malan, T. P., Hruby, V. J. & Porreca, F. (2004). * J. Neurobiol.* **61**, 126–148. 10.1002/neu.20091 15362157

[bb8] Raffa, R. B. (2001). *J. Clin. Pharm. Ther.* **26**, 257–264. 10.1046/j.1365-2710.2001.00355.x 11493367

[bb9] Sheldrick, G. M. (2008). *Acta Cryst.* A**64**, 112–122. 10.1107/S0108767307043930 18156677

